# New Advances in Ovarian Cancer

**DOI:** 10.3390/biomedicines14102247

**Published:** 2026-10-04

**Authors:** Zhenshan Yang, Hengrui Liu

**Affiliations:** 1 Reproductive Medicine Center, Renmin Hospital, Biomedical Engineering College, Hubei University of Medicine, Shiyan 442000, China; 2 Hubei Clinical Research Center for Reproductive Medicine, Shiyan 442000, China; 3 Shiyan Key Laboratory of Reproduction and Genetics, Renmin Hospital, Hubei University of Medicine, Shiyan 442000, China; 4 Hubei Key Laboratory of Embryonic Stem Cell Research, Hubei University of Medicine, Shiyan 442000, China; 5 Department of Biochemistry, University of Cambridge, Cambridge CB2 1QX, UK

## 1. Introduction

Ovarian cancer remains one of the most challenging malignancies in gynecologic oncology, with ongoing efforts to improve early detection, treatment strategies, and patient outcomes [1]. High-grade serous ovarian cancer (HGSOC) is the most common subtype of epithelial ovarian cancer, which accounts for around 70% of all cases [2]. Less common epithelial subtypes include endometrioid, clear cell, low-grade serous, mucinous, and carcinosarcoma [3]. Ovarian cancer is often diagnosed in most patients when it is already at an advanced stage due to a lack of specific symptoms. Standard management, particularly for advanced epithelial ovarian cancer, is based on cytoreductive surgery and platinum-taxane chemotherapy, with maintenance strategies such as anti-angiogenic therapy and/or PARP inhibitors selected according to clinical and molecular features. Long-term survival remains poor. The overall 10-year survival rate of ovarian cancer is <50% today, with outcomes varying substantially by stage and histological subtype. Consequently, it is critical to develop new treatment methods for patients.

Around 50% of HGSOC cases are characterized by a deficiency in homologous recombination (HR), which is one of the main cellular pathways for repairing DNA double-strand breaks [4]. The use of poly (ADP-ribose) polymerase inhibitors (PARPi), which block single-strand DNA damage repair, is synthetically lethal with HR deficiency (HRD) and significantly improves the outcome of patients with HGSOC, especially in those with BRCA1/2-deficient tumors. However, the majority of patients show treatment resistance following the chemotherapy and PARPi treatments, and the five-year recurrence-free survival rate is less than 50% [5]. Therefore, identifying new therapeutic targets is essential to overcome the disease. The tumor immune microenvironment has been increasingly recognized as a critical contributor to therapeutic resistance in ovarian cancer. A recent study suggests that peripheral serotonin activates a subset of tumor-associated macrophages, enhancing HR repair via extracellular vesicle-mediated metabolic crosstalk and thereby promoting chemoresistance. Targeting peripheral serotonin disrupts these pathways and resensitizes tumors to platinum and PARPi therapies, highlighting a novel strategy to overcome therapeutic resistance [6].

PARPi also shows effectiveness in certain tumors that lack BRCA mutations, suggesting alternative mechanisms of action. A recent study showed that PARPi efficacy in BRCA wild-type tumors can be enhanced by telmisartan, an FDA-approved antihypertensive drug that depletes tumor PD-L1 and activates STING-dependent type I interferon signaling [7]. The study shows that inhibition of G2/M checkpoint kinases (PLK1 or WEE1) induces DNA damage and mitotic catastrophe in those HR-proficient HGSOC cells. These preclinical findings provide a rationale for further evaluation of mitotic regulators as therapeutic vulnerabilities in HR-proficient ovarian cancer [8].

Although immunotherapies such as immune checkpoint inhibitors have shown remarkable success in several malignancies, their efficacy in metastatic ovarian cancer remains limited. To address this challenge, recent advances in nanotechnology have enabled the development of innovative delivery systems to enhance local immune activation. In this context, engineered IL-12-polymer-coated liposomal nanoparticles have been designed to anchor and sustain the release of IL-12 within the tumor nodules. This approach promotes robust T cell accumulation in both ascites and tumor tissues in mouse models, significantly prolongs survival, and enhances responsiveness to immune checkpoint inhibitors. These findings highlight the potential of biomaterial-based cytokine delivery systems to overcome the current limitations of immunotherapy and improve therapeutic outcomes in metastatic ovarian cancer [9].

Recent studies have highlighted the critical role of the stromal microenvironment in ovarian cancer initiation. Notably, an epigenetically altered mesenchymal stem cell population, termed high-risk mesenchymal stromal/stem cells (hrMSCs), has been shown to arise prior to the formation of serous tubal intraepithelial carcinoma lesions. hrMSCs promote DNA damage in fallopian tube epithelial (FTE) cells while simultaneously supporting their survival, thereby facilitating malignant transformation and metastatic progression in vivo. Furthermore, the enrichment of hrMSCs in BRCA1/2 mutation carriers and their increase with age suggest a potential link between aging and microenvironmental alterations in ovarian cancer initiation. Collectively, these findings suggest that an altered fallopian tube stromal niche may contribute to HGSOC initiation, although further studies are required to establish its causal and translational significance in humans [10].

**Figure 1:**
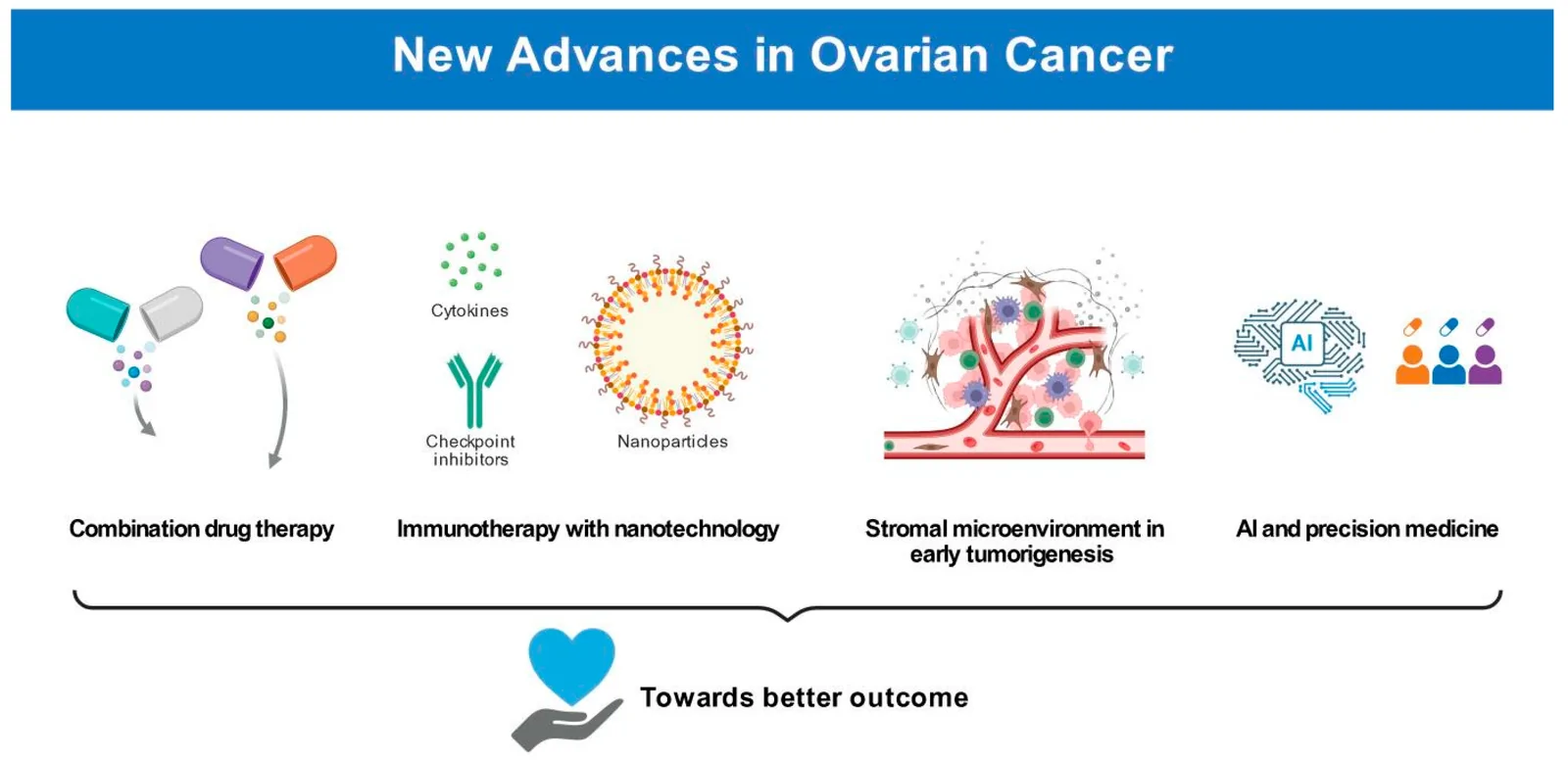
New Advances in Ovarian Cancer. Created in BioRender. Yang, Z. (2027) https://BioRender.com/jdhybbd, access date: 8 July 2026.

Artificial intelligence (AI) has rapidly emerged as a transformative tool in oncology, offering new opportunities for precision medicine in ovarian cancer. Lin et al. systematically summarized the current applications of AI, particularly in the field of radiological imaging, where radiomics and deep learning-based models have demonstrated considerable potential in improving diagnostic accuracy and prognostic assessment. By enabling the extraction and integration of complex imaging features, these approaches may significantly enhance individualized patient management. Nevertheless, the clinical translation of AI technologies remains constrained by several critical challenges, including limited interpretability, concerns regarding generalizability, and unresolved ethical and regulatory issues. Addressing these limitations will be essential for bridging the gap between technological innovation and real-world clinical application (Figure 1) [11].

This Editorial refers to the Special Issue entitled “New Advances in Ovarian Cancer,” which highlights emerging opportunities and challenges in improving the diagnosis, treatment strategies, and clinical outcomes of patients with ovarian cancer. A total of 12 manuscripts were submitted for consideration, all of which underwent a rigorous peer-review process. Ultimately, six papers were accepted for publication in this Special Issue, including five original research articles and one review article.

## 2. An Overview of Published Articles

Fonseca et al. (Contribution 1) explored the potential of drug repurposing as a strategy to overcome chemoresistance in ovarian cancer, focusing on galanthamine, an acetylcholinesterase inhibitor commonly used in Alzheimer’s disease. Given previous suggestions that galanthamine may act as an inhibitor of P-glycoprotein (P-gp), an efflux transporter involved in drug resistance, the authors investigated whether it could reverse paclitaxel resistance in HGSOC cell lines. However, their findings demonstrated that galanthamine neither exerted cytotoxic effects nor enhanced the efficacy of paclitaxel. These results suggest that, despite its theoretical promise, galanthamine is not a viable candidate for overcoming P-gp-mediated chemoresistance in ovarian cancer. Dedes et al. (Contribution 2) provided a comprehensive review of the current understanding of epigenetic modifications in ovarian cancer and their potential clinical implications. The authors highlight the multifaceted role of epigenetic modifications—including DNA methylation, histone modifications, non-coding RNAs, chromatin remodeling, and RNA methylation—in driving tumor initiation, progression, and therapeutic resistance. They further emphasize that distinct epigenetic landscapes across ovarian cancer subtypes offer opportunities for the development of novel biomarkers for early detection, prognosis, and treatment monitoring. Importantly, the review discusses the therapeutic potential of targeting epigenetic regulators, such as DNMT and HDAC inhibitors, as well as EZH2 antagonists, to overcome chemoresistance and enhance responses to immunotherapy.

In the third article, Güler (Contribution 3) focuses on the combined therapeutic potential of hesperidin, a natural bioflavonoid, and adriamycin in ovarian cancer cells through an integrated experimental and bioinformatic approach. Using SKOV3 cells, the authors demonstrated that co-treatment exerted synergistic cytotoxicity effects, significantly enhancing apoptosis compared to either agent alone. Mechanistically, this combination upregulated pro-apoptotic markers, including Caspase-3 and Bax, while downregulating FOXP3 and EGFR. These in vitro findings in SKOV3 cells suggest that combining natural compounds with chemotherapeutic agents may warrant further validation in additional ovarian cancer models. Pappa et al. (Contribution 4) conducted a retrospective study to characterize the clinicopathological features and oncologic outcomes of patients with endometrioid ovarian carcinoma. Their findings indicate that this subtype is frequently diagnosed at an early stage and is commonly associated with low-grade carcinomas, contributing to favorable survival outcomes. Notably, the presence of residual disease and advanced stages are independently associated with inferior survival outcomes. In contrast, lymph node dissection and adjuvant chemotherapy did not significantly impact overall and recurrence-free survival in this cohort. These results may indicate the importance of improved molecular stratification to guide personalized treatment strategies in endometrioid ovarian cancer, although findings should be interpreted cautiously and require further validation.

The article by Gacuta et al. (Contribution 5) is also focused on endometrioid ovarian cancer. They investigated the diagnostic potential of selected matrix metalloproteinases (MMPs) as novel biomarkers for endometrioid ovarian cancer. In this pilot study, the authors demonstrated that several metalloproteinases, particularly MMP-7, MMP-10, MMP-11, and MMP-26, were significantly elevated in patients with endometrioid ovarian cancer compared to healthy women. Among them, MMP-7 exhibited the highest diagnostic performance. These findings suggest that specific metalloproteinases may serve as promising serologic markers for the diagnosis of endometrioid ovarian cancer, complementing existing markers such as CA125 and HE4. However, further validation in larger cohorts is required to confirm their clinical utility.

Colomar-Roig et al. (Contribution 6) assessed a locally implemented semi-automated HRD testing workflow using the Agilent SureSelect CD HRR17 panel with SeqOne/SomaHRD analysis in HGSOC and compared its performance with the commercially established Myriad myChoice CDx and SOPHiA DDM Dx HRD Solution. The results demonstrated high concordance in HRD classification and genomic instability metrics across platforms, with strong correlations between genomic instability metrics and fewer inconclusive results observed in the Agilent/SeqOne workflow. Discrepancies were mainly limited to borderline cases near classification thresholds. These findings indicate that the local workflow provides a reliable, flexible, and practical alternative to commercial HRD testing, supporting its potential application in the diagnosis of ovarian cancer patients.

## 3. Conclusions

In summary, the contributions included in this Special Issue collectively highlight the multifaceted nature of ovarian cancer, spanning molecular mechanisms, therapeutic strategies, biomarker discovery, and clinical management. Despite significant advances, ovarian cancer remains a highly heterogeneous disease with persistent challenges in early diagnosis, treatment resistance, and long-term patient outcomes. Continued progress in understanding the complex biology of ovarian cancer will be critical for developing more effective diagnostic tools and therapeutic strategies, with the goal of improving survival and quality of life for patients.

## References

[1] Scott C.L., Banerjee S., Joly F., Lee J.M., Mukhopadhyay A., Tan D.S., Kohn E.C. (2026). Ovarian cancer. Nat. Rev. Dis. Primers.

[2] Kordowitzki P., Lange B., Elias K.M., Haigis M.C., Mechsner S., Braicu I.E., Sehouli J. (2025). Transforming treatment paradigms: Focus on personalized medicine for high-grade serous ovarian cancer. CA Cancer J. Clin..

[3] Caruso G., Weroha S.J., Cliby W. (2025). Ovarian Cancer: A Review. JAMA.

[4] Chiappa M., Guffanti F., Bertoni F., Colombo I., Damia G. (2021). Overcoming PARPi resistance: Preclinical and clinical evidence in ovarian cancer. Drug Resist. Updat..

[5] Banerjee S., Moore K.N., Colombo N., Scambia G., Kim B.G., Oaknin A., Friedlander M., Lisyanskaya A., Floquet A., Leary A. (2021). Maintenance olaparib for patients with newly diagnosed advanced ovarian cancer and a BRCA mutation (SOLO1/GOG 3004): 5-year follow-up of a randomised, double-blind, placebo-controlled, phase 3 trial. Lancet Oncol..

[6] Li J., Lu J., Zheng C., Huang X., Li H., Mai Q., Chen S., Zhou Z., Zhu J., Yu T. (2026). Serotonin-licensed macrophages potentiate chemoresistance via inositol metabolic crosstalk in ovarian cancer. Cell Metab..

[7] Murray C.E., Ontiveros C.O., Wentworth J., Blinkiewicz P., Leung B., Bai H., Spicer N., Holtz A., Tanner C., Balasubramanian A. (2026). Telmisartan increases olaparib efficacy in homologous recombination proficient tumors by augmenting type I interferon production. J. Immunother. Cancer.

[8] Xi Q., Kunita A., Ogawa M., Kawakami M., Ka M., Nagai S., Duong A.Q., Taguchi A., Watanabe K., Fukuda T. (2025). PLK1 or WEE1 inhibition targets homologous recombination repair proficiency in BRCA1/2 wild-type high-grade serous ovarian cancer. Cell Death Dis..

[9] Pires I.S., Covarrubias G., Gomerdinger V.F., Backlund C., Nombera Bueno E., Billingsley M.M., Pryor M., Shanker A., Gordon E., Wu S. (2026). IL-12-releasing nanoparticles for effective immunotherapy of metastatic ovarian cancer. Nat. Mater..

[10] Garcia G.L., Orellana T., Gorecki G., Frisbie L., Baruwal R., Suresh S., Goldfeld E., Beddows I., MacFawn I.P., Britt A.K. (2025). Aged and BRCA-Mutated Stromal Cells Drive Epithelial Cell Transformation. Cancer Discov..

[11] Lin Z., Qiang J., Gu Y., Li H. (2025). Artificial Intelligence in Ovarian Cancer: Current Advances and Perspectives. Med. Adv..

